# Post-mastectomy Radiotherapy in T1-2 Breast Cancer Patients With One to Three Lymph Node Metastases: A Propensity Score Matching Analysis

**DOI:** 10.3389/fonc.2019.01551

**Published:** 2020-02-14

**Authors:** Maoshan Chen, Yunhui Huang, Zhengwei Leng, Guanglun Yang, Fangfang Li, Hongwei Yang, Lingmi Hou

**Affiliations:** ^1^Department of Breast Surgery, Suining Central Hospital, Suining, China; ^2^Department of Breast and Thyroid Surgery, Hepatobiliary and Pancreatic Institution, Affiliated Hospital of North Sichuan Medical College, Nanchong, China; ^3^Department of Endocrine and Breast Surgery, First Affiliated Hospital of Chongqing Medical University, Chongqing, China; ^4^Department of Operating Room, Suining Central Hospital, Suining, China

**Keywords:** breast neoplasms, lymph node, radiotherapy, prognosis, Surveillance, Epidemiology, and End Results (SEER) Program

## Abstract

**Background and Objectives:** Whether post-mastectomy radiotherapy (PMRT) could improve prognosis for T1-2 breast cancer patients with one to three lymph node metastases remains controversial. The present study aimed to determine the significance of PMRT in patients with T1-2N1M0 breast cancer.

**Methods:** Data of 45,646 patients from the Surveillance, Epidemiology, and End Results (SEER) database were analyzed; 12,585 matched patients were divided into a PMRT group and non-radiotherapy group (no-PMRT), respectively, using the propensity score matching method. Univariate and multivariate analyses were performed to determine the prognostic factors of breast cancer, and subgroup analysis was performed according to the number of lymph node metastases.

**Results:** With the median follow-up of 62 months, 5-year cancer-specific survival was 91.48% in the PMRT group and 91.88% in the no-PMRT group (*P* = 0.405). PMRT did not improve the breast cancer-specific survival (BCSS) in patients with stage T1-2N1M0 (HR = 0.99, 95% CI = 0.92–1.06, *P* = 0.715). In subgroup analysis, radiotherapy improved the BCSS in patients with three nodes positive, with the 5-year BCSS at 88.5% in the radiation group and 86.6% in the no-radiation group (HR = 0.78, 95% CI = 0.65–0.90, *P* < 0.001). In patients with two nodes positive, 5-year BCSS was 90.3% in the PMRT group and 89.5% in the no-PMRT group, with no significant difference between the two groups (HR = 0.96, 95% CI = 0.85–1.09, *P* = 0.552). In patients with one node positive, 5-year BCSS was higher in the no-PMRT group (92.1%) than that in the PMRT group (90.8%); radiotherapy increased the cancer-related death compared with those who did not receive it (HR = 1.21, 95% CI = 1.08–1.36, *P* = 0.002).

**Conclusion:** The benefit of PMRT in T1-2N1M0 patients was obviously different, and the recommendation of PMRT for this population should be individualized. PMRT should be considered for patients with three nodes positive, should be suggested cautiously in those with two nodes positive, and could be omitted in those with one node positive.

## Introduction

The incidence of breast cancer ranks first among all the female malignant tumors and it has become the second largest reason for females' deaths (1). Breast cancer screening and improvement of comprehensive treatment have significantly improved the prognosis for breast cancer patients; however, better prognosis would be necessarily relying on further development of comprehensive treatments (2, 3). In the era of precision medicine, individualized treatment strategies are formulated according to the patient's tumor stage, recurrence risk, and treatment sensitivity (4). Axillary lymph node status is not only an important reference for accurate staging of breast cancer but also an important index for evaluating prognosis and guiding treatment (2, 5, 6). And the number of lymph node metastases is closely related to prognosis (5–7). It is proven by a series of clinical studies that combined radiotherapy after mastectomy can improve the survival of patients with four or more positive lymph nodes (8–11).

However, whether post-mastectomy radiotherapy (PMRT) could improve prognosis for T1-2 breast cancer patients with one to three lymph node metastases remains controversial (8, 11–13). In EBCTCG meta-analysis, 1,314 patients with one to three axillary lymph node metastases were analyzed, and the results showed that PMRT could reduce local recurrence and improve overall survival (11). The sample size of this study was small, and cases dated far back in time (1964–1986), when the local radiotherapy technology and systemic treatment were too underdeveloped to guide treatments under modern medical conditions. A recent study reported by the University of Chicago showed that radiotherapy improved the prognosis of patients with two lymph nodes positive with 2–5 cm size of tumors and patients with three lymph nodes positive (14). Nevertheless, Muhsen and colleagues analyzed 1,087 patients with T1-2N1 breast cancer to investigate the value of PMRT (15). The results showed that PMRT could not improve the recurrence-free survival and overall survival. A series of studies had evaluated the significance of PMRT in T1-2N1 breast cancer patients, and the conclusions were discordant (8, 11, 14, 16–18).

With the available evidence, the recommendations of PMRT for T1-2N1M0 breast cancer were quite discrepant (4, 19, 20). Considering the limitations of these studies, such as small sample and low level of evidence, recommending clinicians and patients should balance the benefit and radiation toxicity. It is necessary to evaluate the value of PMRT in T1-2 breast cancer with one to three nodes' metastases and to explore the predictor for choice of PMRT.

In this study, we used real-world data from the American Surveillance, Epidemiology, and End Results (SEER) database to investigate the prognostic value of PMRT for T1-2N1M0 breast cancer patients. Simultaneously, we conducted a subgroup analysis to determine which patients were suitable to receive PMRT to assist physicians with clinical decision making for this controversial group.

## Materials and Methods

### Data Source and Patient Selection

This retrospective cohort study analyzed de-identified data from the SEER 18 Registry Research Data set, which collects data on patients' demographics, tumor characteristics, first course of treatment, and follow-up for vital status. The case listing in this retrospective cohort study was generated by SEER^*^Stat software (version 8.3.5). Female patients with the first primary stages of T1-2N1M0 breast cancer diagnosed between 2000 and 2014 were selected from the SEER database. We identified potentially eligible patients based on the following inclusion criteria: female, year of diagnosis between the years 2000 and 2014, unilateral breast cancer, pathological confirmation of invasive carcinoma, breast cancer as the first and only malignant cancer diagnosis, having received a mastectomy (including breast and axillary lymph nodes) with or without reconstruction, tumor stage T1 or T2, one to three lymph node metastases, and no distance metastasis at diagnosis. Patients were excluded for missing any information such as radiotherapy record, age at diagnosis, surgery record, chemotherapy record, estrogen receptor (ER) status, progesterone receptor (PR) status, grade, tumor stage, marital status, survival status, and time. Besides, patients who received radiotherapy other than post-mastectomy were excluded as well. The final study sample contained 45,646 patients. Histologic types of tumors are classified according to the International Classification of Disease for Oncology (ICD-O), 3rd Edition. Tumor stage is categorized according to the American Joint Committee on Cancer (AJCC) staging system, 6th edition. Breast cancer subtypes were classified into HR+/HER2–, HR+/HER2+, HR–/HER2+, and HR–/HER2– according to the status of the hormone receptor (HR) and human epidermal receptor 2 (HER2).

### Clinicopathologic Parameters

The following variables were collected: year of diagnosis, age at diagnosis, race, marital status, laterality, histology, tumor size, AJCC stage, surgery procedure, number of regional lymph nodes examined, number of regional lymph nodes positive, histological grade, ER status, PR status, chemotherapy recode, radiotherapy recode, follow-up, and vital status.

In this study, breast cancer-specific survival (BCSS) was used as the primary study outcome, indicating the survival time between the dates of diagnosis and the date of death due to breast cancer. Patients who died of other causes were censored on the data of death.

### Statistical Analyses

Clinicopathologic features were compared between the radiotherapy (PMRT) group and no-radiotherapy (no-PMRT) group using Pearson's chi-square test. To balance of baseline characteristics between each group, propensity score matching (PSM) analysis was conducted with a ratio of 1.0. BCSS was estimated, and the survival curves were plotted using the Kaplan-Meier method. The log-rank test was used to identify prognostic factors for BCSS. The hazard ratios (HRs) with 95% confidence intervals (CIs) for BCSS of patients in the PMRT group compared with patients in the no-PMRT group were evaluated via univariate and multivariate Cox regression models. Parameters with a statistical significance in univariate analysis or with a clinical significance were included in the multivariate Cox model. Adjusted HRs and 95% CIs were calculated using multivariate Cox proportion hazard models, with adjustment for age at diagnosis, race, marital status, tumor stage, number of nodes positive, ER status, PR status, grade, and chemotherapy. Patients were classified into the one, two, or three nodes positive subgroup according to the number of lymph node metastases. Multivariate Cox models were conducted in subgroup analyses to determine whether there was a significant interaction between different features and radiotherapy in predicting BCSS. Statistical analyses were performed using Stata software (version 13.0 SE). A two-side *P* value lower than 0.05 was deemed to indicate statistical significance.

## Results

### Demographic and Tumor Characteristics

A total of 45,646 patients with primary invasive breast cancer who met the study criteria were eventually selected. The median age of all patients was 52 years (IQR: 44–62). Patients' demographics and tumor characteristics stratified by radiotherapy are summarized in Table 1. There were significant differences in all variables between the PMRT group and no-PMRT group (*P* < 0.001). The PSM method was used to balance baseline features between each group, and there were 12,585 patients in each group after PSM. Between the two groups, the number of lymph nodes positive showed a statistically significant difference (*P* < 0.001); for other baseline characteristics, no significant differences were observed (*P* > 0.05, Table 1).

**Table 1:** Comparison of demographic and tumor characteristics between PMRT group and no-PMRT group.

**Variables**	**Before PSM**		***P*-value**	**After PSM**		***P*-value**
	**PMRT** ***N* = 12,585 (%)**	**No-PMRT** ***N* = 33,061 (%)**		**PMRT** ***N* = 12,585 (%)**	**No-PMRT** ***N* = 12,585 (%)**	
Age (years)						
<40	1,885 (14.98)	2,687 (8.13)	<0.001	1,885 (14.98)	1,754 (13.94)	0.053
40–70	9,261 (73.59)	22,517 (68.11)		9,261 (73.59)	9,344 (74.25)	
≥70	1,439 (11.43)	7,857 (23.77)		1,439 (11.43)	1,487 (11.82)	
Race						
White	9,782 (77.73)	26,603 (80.47)	<0.001	9,782 (77.73)	9,803 (77.89)	0.703
Black	1,483 (11.78)	3,355 (10.15)		1,483 (11.78)	1,443 (11.47)	
Other^*^	1,320 (10.49)	3,103 (9.39)		1,320 (10.49)	1,339 (10.64)	
Marital status						
Married	8,187 (65.05)	19,778 (59.82)	<0.001	8,187 (65.05)	8,193 (65.10)	0.937
Unmarried^#^	4,398 (34.95)	13,283 (40.18)		4,398 (34.95)	4,392 (34.90)	
Tumor stage						
T1	4,188 (33.28)	15,293 (46.26)	<0.001	4,188 (33.28)	4,176 (33.18)	0.872
T2	8,397 (66.72)	17,768 (53.74)		8,397 (66.72)	8,409 (66.82)	
Grade						
1	1,151 (9.15)	4,606 (13.93)	<0.001	1,151 (9.15)	1,149 (9.13)	0.965
2	5,261 (41.80)	14,973 (45.29)		5,261 (41.80)	5,242 (41.65)	
3^†^	6,173 (49.05)	13,482 (40.78)		6,173 (49.05)	6,194 (49.22)	
ER status						
Negative	2,854 (22.68)	6,417 (19.41)	<0.001	2,854 (22.68)	2,806 (22.30)	0.469
Positive	9,731 (77.32)	26,644 (80.59)		9,731 (77.32)	9,779 (77.70)	
PR status						
Negative	4,218 (33.52)	9,972 (30.16)	<0.001	4,218 (33.52)	4,191 (33.30)	0.718
Positive	8,367 (66.48)	23,089 (69.84)		8,367 (66.48)	8,394 (66.70)	
No. of LNs positive						
One	5,590 (44.42)	20,316 (61.45)	<0.001	5,590 (44.42)	5,545 (44.06)	<0.001
Two	3,923 (31.17)	8,647 (26.15)		3,923 (31.17)	4,276 (33.98)	
Three	3,072 (24.41)	4,098 (12.40)		3,072 (24.41)	2,764 (21.96)	
Chemotherapy						
No	1,591 (12.64)	13,709 (41.47)	<0.001	1,591 (12.64)	1,592 (12.65)	0.985
Yes	10,994 (87.36)	19,352 (58.53)		10,994 (87.36)	10,993 (87.35)	
Specific death	1,375 (10.93)	3,588 (10.85)	–	1,375 (10.93)	1,542 (12.25)	–

* *Including American Indian/AK Native, and Asian/Pacific Islander*.

# *Including single, separated, divorced, widowed, and unmarried or domestic partner. †Including poorly differentiated and undifferentiated. PMRT, post-mastectomy radiotherapy; ER, estrogen receptor; PR, progesterone receptor; LN, lymph node*.

### Association of PMTR and BCSS

**Figure 1:**
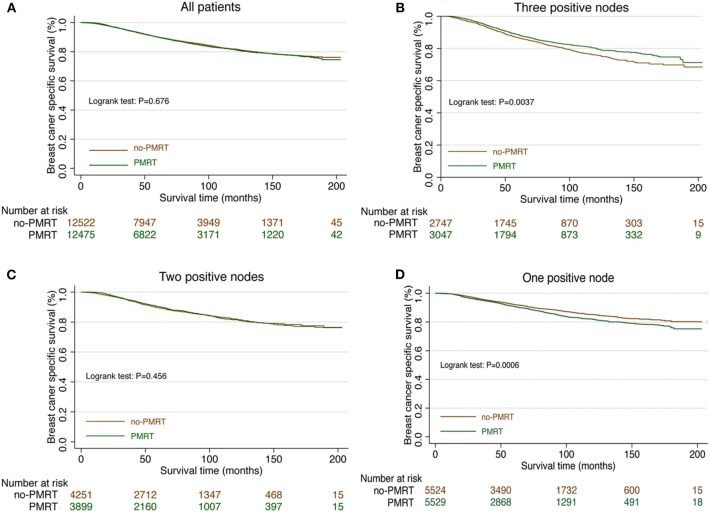
Survival curve in T1-2N1M0 breast cancer patients with and without PMRT. **(A)** The survival curve in all T1-2N1M0 breast cancer patients with PMRT and no PMRT. **(B)** The survival curve in T1-2 and three nodes positive breast cancer patients with PMRT and no PMRT. **(C)** The survival curve in T1-2 and two nodes positive breast cancer patients with PMRT and no PMRT. **(D)** The survival curve in T1-2 and one node positive breast cancer patients with PMRT and no PMRT.

Median follow-up was 62 months (IQR: 29–107), and 2,917 women died of breast cancer. There were 1,375 (10.93%) breast cancer-related death events observed in the PMRT group and 1,542 (12.25%) in the no-PMRT group. The 5-year cancer-specific survival was 91.48% in the PMRT group and 91.88% in the no-PMRT group (*P* = 0.405). Kaplan-Meier analysis showed that patients who received PMRT had a similar BCSS compared with patients who did not receive PMRT; the log-rank test *P* value was 0.676 (Figure 1A). In univariate analysis, age, race, marital status, tumor stage, grade, ER status, PR status, and number of lymph node metastases were significantly associated with BCSS. However, chemotherapy was not associated with BCSS. For clinical consideration, all variables associated with BCSS and chemotherapy were included in the multivariate Cox regression model. The multivariate analysis results were almost consistent with the result of the univariate analysis except chemotherapy; the details are shown in Table 2. In multivariate Cox regression analysis, radiotherapy did not significantly improve the cancer-specific survival for breast cancer patients (HR = 0.99, 95% CI = 0.92–1.06, *P* = 0.715, Table 2).

**Table 2:** Univariate and multivariate analysis for BCSS in patients with one to three LNs positive.

**Variable**	**Univariate analysis**		**Multivariate analysis**	
	**HR** **(95% CI)**	***P***	**HR** **(95% CI)**	***P***
Age at diagnosis (years)				
<40	Reference	–	Reference	–
40–70	0.77 (0.70–0.85)	<0.001	0.84 (0.76–0.93)	0.001
≥70	1.14 (1.07–1.22)	<0.001	1.14 (1.06–1.24)	0.001
Race				
White	Reference	–	Reference	–
Black	1.52 (1.38–1.69)	<0.001	1.23 (1.11–1.37)	<0.001
Other	0.96 (0.90–1.02)	0.167	0.96 (0.90–1.02)	0.186
Marital status				
Married	Reference	–	Reference	–
Unmarried	1.30 (1.21–1.40)	<0.001	1.24 (1.15–1.34)	<0.001
Tumor stage				
T1	Reference	–	Reference	–
T2	1.88 (1.72–2.05)	<0.001	1.68 (1.54–1.83)	<0.001
Grade				
1	Reference	–	Reference	–
2	2.07 (1.67–2.55)	<0.001	1.77 (1.43–2.19)	<0.001
3	2.01 (1.81–2.22)	<0.001	1.70 (1.53–1.89)	<0.001
ER status				
Negative	Reference	–	Reference	–
Positive	0.43 (0.40–0.46)	<0.001	0.71 (0.63–0.79)	<0.001
PR status				
Negative	Reference	–	Reference	–
Positive	0.45 (0.42–0.48)	<0.001	0.68 (0.61–0.76)	<0.001
No. of LNs positive				
1	Reference	–	Reference	–
2	1.14 (1.04–1.24)	0.004	1.13 (1.03–1.23)	0.007
3	1.19 (1.14–1.24)	<0.001	1.19 (1.14–1.25)	<0.001
Chemotherapy				
No	Reference	–	Reference	–
Yes	0.92 (0.82–1.03)	0.130	0.77 (0.68–0.86)	<0.001
Radiotherapy				
No	Reference	–	Reference	–
Yes	1.02 (0.94–1.09)	0.676	0.99 (0.92–1.06)	0.715

*BCSS, breast cancer–specific survival*.

### Survival Analysis for Subgroups

**Figure 2:**
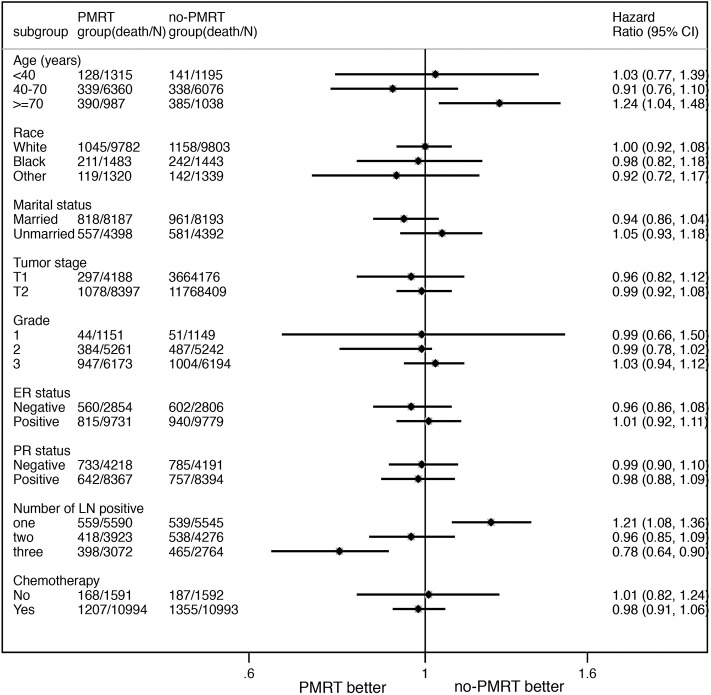
The forest plot for HR comparing BCSS between the PMRT group and no-PMRT group according to different variables.

Subgroup analyses using the Cox model were conducted to further determine the effect of radiotherapy on BCSS in patients with different features. The baseline characteristic features between the PMRT group and no-PMRT group were almost balanced in patients with one, two, or three positive nodes (Supplementary Tables 1–3). Radiotherapy was not associated with improved BCSS in patients with age <70 years. In contrast, patients over 70 years who received PMRT had an adverse impact on BCSS (HR = 1.24, 95% CI = 1.04–1.48, *P* = 0.017, Table 3). In the setting with three lymph nodes positive, the 5-year cancer-specific survival was 88.5% in the radiation group and 86.6% in the no-radiation group. Radiotherapy improved the BCSS in patients with three nodes positive (HR = 0.78, 95% CI = 0.65–0.90, *P* < 0.001, Figure 1B, Table 3). In the setting with two lymph nodes positive, the 5-year cancer-specific survival was 90.3% in the radiation group and 89.5% in the no-radiation group. There was no significant difference of survival in patients with two lymph node metastases between the PMRT group and no-PMRT group (HR = 0.96, 95% CI = 0.85–1.09, *P* = 0.552, Figure 1C, Table 3). Surprisingly, we found that the 5-year cancer-specific survival in patients with one lymph node positive was higher in the no-PMRT group (92.1%) than that of the PMRT group (90.8%). Radiotherapy increased the cancer-related death compared with those who did not receive it (HR = 1.21, 95% CI = 1.08–1.36, *P* = 0.002, Figure 1D, Table 3). All subgroup analyses are summarized in Table 3 and shown in Figure 2.

**Table 3:** Subgroup analyses of radiotherapy effect on BCSS in patients with different features.

**Variable**	**PMRT group death/patient**	**No-PMRT group death/patient**	**HR (95% CI)**	***P***
Age				
<40	128/1,315	141/1,195	1.03 (0.77–1.39)	0.831
40–70	339/6,360	338/6,076	0.91 (0.76–1.10)	0.338
≥70	390/987	385/1,038	1.24 (1.04–1.48)	0.017
Race				
White	1,045/9,782	1,158/9,803	1.00 (0.92–1.08)	0.937
Black	211/1,483	242/1,443	0.98 (0.82–1.18)	0.858
Other	119/1,320	142/1,339	0.92 (0.72–1.17)	0.485
Marital status				
Married	818/8,187	961/8,193	0.94 (0.86–1.04)	0.223
Unmarried	557/4,398	581/4,392	1.05 (0.93–1.18)	0.423
Tumor stage				
T1	297/4,188	366/4,176	0.96 (0.82–1.12)	0.607
T2	1,078/8,397	1,176/8,409	0.99 (0.92–1.08)	0.911
Grade				
1	44/1,151	51/1,149	0.99 (0.66–1.50)	0.981
2	384/5,261	487/5,242	0.99 (0.78–1.02)	0.105
3	947/6,173	1,004/6,194	1.03 (0.94–1.12)	0.536
ER status				
Negative	560/2,854	602/2,806	0.96 (0.86–1.08)	0.482
Positive	815/9,731	940/9,779	1.01 (0.92–1.11)	0.779
PR status				
Negative	733/4,218	785/4,191	0.99 (0.90–1.10)	0.914
Positive	642/8,367	757/8,394	0.98 (0.88–1.09)	0.725
No. of LNs positive				
1	559/5,590	539/5,545	1.21 (1.08–1.36)	0.002
2	418/3,923	538/4,276	0.96 (0.85–1.09)	0.552
3	398/3,072	465/2,764	0.78 (0.64–0.90)	<0.001
Chemotherapy				
No	168/1,591	187/1,592	1.01 (0.82–1.24)	0.955
Yes	1,207/10,994	1,355/10,993	0.98 (0.91–1.06)	0.617

## Discussion

In this study, we used real-world data from the SEER database to investigate the impact of PMRT on BCSS by the PSM method in breast cancer patients with stage T1-2N1M0. There was a similar 5-year cancer-specific survival rate in patients with or without PMRT; the percentage was 90.1% in the PMRT group and the 90.0% in the no-PMRT group. For women with stage T1-2 and three lymph node metastases, radiotherapy after mastectomy could improve the BCSS. For patients with one or two lymph node metastases, radiotherapy did not bring specific survival benefits.

Lymph node status is an important indicator of prognosis and treatment. The number of node metastases reflected the tumor burden; the more node metastases, the higher the recurrence risk (5, 6). PMRT was an important technique to reduce the recurrence risk, and a series of studies had demonstrated that radiotherapy could improve the survival in patients with four or more lymph node metastases (11, 21, 22). However, the recommendations of local radiotherapy for stage T1-2 patients with one to three lymph nodes positive were obviously different (4, 19, 20). The EBCTCG meta-analysis and DBCG 82b&c study demonstrated that PMRT could reduce the local recurrence and improve survival (8, 11). The trials included by the EBCTCG were predominantly conducted in the 1970s and 1980s. The local recurrence rate at 10 years (21%) reported in this meta-analysis was considerably higher than that (4~10%) reported in the later series (20). Otherwise, previous studies had a small sample size, and the limitations of these studies' design decreased the evidence grade. Therefore, this evidence coming from elder circumstances was not suitable to guide treatment in the modern medicine era (20). The conclusions of whether PMRT could improve the survival for T1-2N1M0 breast cancer patients were controversial, and which patients benefit from PMRT is unclear. This study found that the number of lymph node metastases was closely related to the benefit of radiotherapy.

In this study, radiotherapy improved the cancer-specific survival in patients with three nodes positive but did not benefit the patients with one or two lymph nodes positive. Patients with three lymph nodes positive have a higher tumor burden and possibly higher risk of recurrence and metastasis than those of patients with one or two lymph nodes positive (6, 23). The escalation of local management allows patients to have a better local control and brings survival benefits to patients with a high recurrence risk. In the Z0011 and AMAROS trails, the 5-year survival rate in the radiotherapy group was significantly higher than that in the non-radiotherapy group in patients with three nodes positive (24, 25). The recurrence rate in patients with one or two nodes positive was mild and moderate, especially under the systemic treatment in modern times (26). McBride and his colleagues investigated the value of radiotherapy in different years and found that patients in the past old times were the major benefitted population (17). Patients with one or two nodes positive who received axillary lymph node dissection or radiotherapy had a similar local recurrence rate and disease-free survival (25, 27). With the appropriate systemic treatment, the benefits of radiotherapy after mastectomy were limited for patients with low recurrence risk. Therefore, PMRT should not be considered routinely for patients with one or two lymph node metastases after mastectomy and axillary dissection.

In addition, the radiotherapy group showed even worse survival benefits among T1-2 breast cancer patients with one lymph node positive. Patients with only one lymph node metastasis usually bear low tumor metastasis load and recurrence rate, and thus could expect relatively good prognosis with systematic treatment (chemotherapy, targeted therapy, and/or endocrine therapy) (26). For patients with a low risk of recurrence, adding radiotherapy after mastectomy may cause an interaction between radiation, tumor cells, and the immune system, which influenced the patients' survival (28, 29). The complications of radiotherapy, such as pneumonitis, lymphedema, and cardiac toxicities, et al. may lead to even worse results (20). A retrospective study analyzed the impact of radiotherapy on survival in patients collected from the NCDB and SEER database between the years 1998 and 2008. It was reported that radiotherapy could not improve the prognosis of patients with one or two lymph node metastases and with tumor size <2 cm (14). Therefore, there is insufficient evidence to recommend PMRT to patients with one lymph node metastasis.

Radiotherapy can bring not only survival benefits but also side effects—lung injury, cardiac, and skin side effects, et al., which decreased the quality of life of patients (30, 31). In the T1-2N1M0 breast cancer population, the benefit of radiotherapy may be less than the side effects in some patients. Clinicopathological features that can effectively predict the benefits of radiotherapy could be helpful for clinical decision making. This study found that the number of lymph node metastases may be a reference parameter to predict the benefit of radiotherapy and avoid the side effects of unnecessary radiation in some patients. Further study is needed to validate this result.

Our study revealed that a positive number of lymph nodes was an important indicator for predicting a benefit from PMRT in stage T1-2N1M0 breast cancer patients. PMRT should be recommended to patients with three lymph node metastases regularly. Whether it is recommended to patients with one or two node metastases should balance the potential benefits and risks. The limitations of our study were that the SEER database did not provide details of the irradiated technique and scope, the absence of local regional recurrence data, and that the molecular subtype information only gotten from the year 2010 resulted in a small sample size and short follow-up time for subgroup analysis. We look forward to the results of the prospective SUPREMO trial, which randomly allocated breast cancer patients with high-risk node-negative disease and those with one to three positive nodes to receive PMRT or not (32). The results of this trial may eventually help determine which patients are most likely to benefit from PMRT when modern systemic treatment and surgery are used.

## Conclusion

The benefit of PMRT in T1-2N1M0 patients was obviously different, and the recommendation of PMRT for this population should be individualized. PMRT should be recommended to patients with three nodes positive, should be suggested cautiously in those with two nodes positive, and could be omitted in those with one node positive.

## Data Availability Statement

Publicly available datasets were analyzed in this study. These data can be found here: www.seer.cancer.gov.

## Ethics Statement

The studies involving human participants were reviewed and approved by the ethical committee of Affiliated Hospital of North Sichuan Medical College (Number: 2018EA030). Written informed consent for participation was not required for this study in accordance with the national legislation and the institutional requirements.

## Author Contributions

LH and HY designed the study. MC, YH, and HY wrote the primary manuscript. FL, YH, and ZL extracted the data from the SEER database. YH, ZL, MC, and GY performed the statistical analysis. All authors read and approved the final manuscript.

### Conflict of Interest

The authors declare that the research was conducted in the absence of any commercial or financial relationships that could be construed as a potential conflict of interest.

## References

[1] Siegel RL Miller KD Jemal A Cancer statistics, 2018. CA Cancer J Clin. (2018) 68:277–300. 10.3322/caac.21442 29313949

[2] Harbeck N Gnant M. Breast cancer. Lancet. (2017) 389:1134–50. 10.1016/S0140-6736(16)31891-8 27865536

[3] Smith RA Andrews KS Brooks D Fedewa SA Manassaram-Baptiste D Saslow D. Cancer screening in the United States, 2017: a review of current American Cancer Society guidelines and current issues in cancer screening. CA Cancer J Clin. (2017) 67:100–21. 10.3322/caac.21392 28170086

[4] Curigliano G Burstein HJ Winer EP Gnant M Dubsky P Loibl S. De-escalating and escalating treatments for early-stage breast cancer: the St. Gallen International Expert Consensus Conference on the Primary Therapy of Early Breast Cancer 2017. Ann Oncol. (2017) 28:1700–12. 10.1093/annonc/mdx308 28838210 PMC6246241

[5] Ribelles N Perez-Villa L Jerez JM Pajares B Vicioso L Jimenez B. Pattern of recurrence of early breast cancer is different according to intrinsic subtype and proliferation index. Breast Cancer Res. (2013) 15:R98. 10.1186/bcr3559 24148581 PMC3978680

[6] Moo TA McMillan R Lee M Stempel M Patil S Ho A. Selection criteria for postmastectomy radiotherapy in t1-t2 tumors with 1 to 3 positive lymph nodes. Ann Surg Oncol. (2013) 20:3169–74. 10.1245/s10434-013-3117-0 23975289

[7] Darby S McGale P Correa C Taylor C Arriagada R Clarke M. Effect of radiotherapy after breast-conserving surgery on 10-year recurrence and 15-year breast cancer death: meta-analysis of individual patient data for 10,801 women in 17 randomised trials. Lancet. (2011) 378:1707–16. 10.1016/S0140-6736(11)61629-2 22019144 PMC3254252

[8] Overgaard M Nielsen HM Overgaard J. Is the benefit of postmastectomy irradiation limited to patients with four or more positive nodes, as recommended in international consensus reports? A subgroup analysis of the DBCG 82 b&c randomized trials. Radiother Oncol. (2007) 82:247–53. 10.1016/j.radonc.2007.02.001 17306393

[9] Tseng YD Uno H Hughes ME Niland JC Wong YN Theriault R. Biological subtype predicts risk of locoregional recurrence after mastectomy and impact of postmastectomy radiation in a large national database. Int J Radiat Oncol Biol Phys. (2015) 93:622–30. 10.1016/j.ijrobp.2015.07.006 26461004

[10] Frasier LL Holden S Holden T Schumacher JR Leverson G Anderson B. Temporal trends in postmastectomy radiation therapy and breast reconstruction associated with changes in national comprehensive cancer network guidelines. JAMA Oncol. (2016) 2:95–101. 10.1001/jamaoncol.2015.3717 26539936 PMC4713236

[11] McGale P Taylor C Correa C Cutter D Duane F Ewertz M. Effect of radiotherapy after mastectomy and axillary surgery on 10-year recurrence and 20-year breast cancer mortality: meta-analysis of individual patient data for 8135 women in 22 randomised trials. Lancet. (2014) 383:2127–35. 10.1016/S0140-6736(14)60488-8 24656685 PMC5015598

[12] Harris JR. Treatment of regional lymph nodes in breast cancer-not recommended for all patients with 1 to 3 positive auxiliary nodes. JAMA Oncol. (2016) 2:991–2. 10.1001/jamaoncol.2016.0222 27253737

[13] Poortmans PM Coles C Bernier J. Treatment of regional lymph nodes in breast cancer-evidence in favor of radiation therapy. JAMA Oncol. (2016) 2:989–90. 10.1001/jamaoncol.2016.0183 27253513

[14] Huo D Hou N Jaskowiak N Winchester DJ Winchester DP Yao K. Use of postmastectomy radiotherapy and survival rates for breast cancer patients with T1-T2 and one to three positive lymph nodes. Ann Surg Oncol. (2015) 22:4295–304. 10.1245/s10434-015-4528-x 25820998

[15] Muhsen S Moo TA Patil S Stempel M Powell S Morrow M Most breast cancer patients with T1-2 tumors and one to three positive lymph nodes do not need postmastectomy radiotherapy. Ann Surg Oncol. (2018) 25:1912–20. 10.1245/s10434-018-6422-9 29564588 PMC5976529

[16] Tam MM Wu SP Perez C Gerber NK. The effect of post-mastectomy radiation in women with one to three positive nodes enrolled on the control arm of BCIRG-005 at ten year follow-up. Radiother Oncol. (2017) 123:10–4. 10.1016/j.radonc.2017.03.001 28341062

[17] McBride A Allen P Woodward W Kim M Kuerer HM Drinka EK. Locoregional recurrence risk for patients with T1,2 breast cancer with 1-3 positive lymph nodes treated with mastectomy and systemic treatment. Int J Radiat Oncol Biol Phys. (2014) 89:392–8. 10.1016/j.ijrobp.2014.02.013 24721590

[18] Ragaz J Olivotto IA Spinelli JJ Phillips N Jackson SM Wilson KS. Locoregional radiation therapy in patients with high-risk breast cancer receiving adjuvant chemotherapy: 20-year results of the British Columbia randomized trial. J Natl Cancer Inst. (2005) 97:116–26. 10.1093/jnci/djh297 15657341

[19] Cardoso F Kyriakides S Ohno S Penault-Llorca F Poortmans P Rubio IT. Early breast cancer: ESMO Clinical Practice Guidelines for diagnosis, treatment and follow-up. Ann Oncol. (2019) 30:1194–220. 10.1093/annonc/mdz189 31161190

[20] Recht A Comen EA Fine RE Fleming GF Hardenbergh PH Ho AY. Postmastectomy radiotherapy: an American Society of Clinical Oncology, American society for radiation oncology, and society of surgical oncology focused guideline update. Ann Surg Oncol. (2017) 24:38–51. 10.1245/s10434-016-5558-8 27646018 PMC5179596

[21] Whelan TJ Julian J Wright J Jadad AR Levine ML. Does locoregional radiation therapy improve survival in breast cancer? A meta-analysis. J Clin Oncol. (2000) 18:1220–9. 10.1200/JCO.2000.18.6.1220 10715291

[22] Early Breast Cancer Trialists' Collaborative Group Favourable and unfavourable effects on long-term survival of radiotherapy for early breast cancer: an overview of the randomised trials. Lancet. (2000) 355:1757–70. 10.1016/S0140-6736(00)02263-7 10832826

[23] Truong PT Olivotto IA Kader HA Panades M Speers CH Berthelet E. Selecting breast cancer patients with T1-T2 tumors and one to three positive axillary nodes at high postmastectomy locoregional recurrence risk for adjuvant radiotherapy. Int J Radiat Oncol Biol Phys. (2005) 61:1337–47. 10.1016/j.ijrobp.2004.08.009 15817335

[24] Giuliano AE Ballman KV McCall L Beitsch PD Brennan MB Kelemen PR. Effect of axillary dissection vs no axillary dissection on 10-year overall survival among women with invasive breast cancer and sentinel node metastasis: the ACOSOG Z0011 (Alliance) randomized clinical trial. JAMA. (2017) 318:918–26. 10.1001/jama.2017.11470 28898379 PMC5672806

[25] Donker M van Tienhoven G Straver M E Meijnen P van de Velde CJ Mansel RE. Radiotherapy or surgery of the axilla after a positive sentinel node in breast cancer (EORTC 10981-22023 AMAROS): a randomised, multicentre, open-label, phase 3 non-inferiority trial. Lancet Oncol. (2014) 15:1303–10. 10.1016/S1470-2045(14)70460-7 25439688 PMC4291166

[26] Sharma R Bedrosian I Lucci A Hwang RF Rourke LL Qiao W. Present-day locoregional control in patients with t1 or t2 breast cancer with 0 and 1 to 3 positive lymph nodes after mastectomy without radiotherapy. Ann Surg Oncol. (2010) 17:2899–908. 10.1245/s10434-010-1089-x 20443145 PMC4324592

[27] Savolt A Peley G Polgar C Udvarhelyi N Rubovszky G Kovacs E. Eight-year follow up result of the OTOASOR trial: the optimal treatment of the axilla - surgery or radiotherapy after positive sentinel lymph node biopsy in early-stage breast cancer: a randomized, single centre, phase III, non-inferiority trial. Eur J Surg Oncol. (2017) 43:672–9. 10.1016/j.ejso.2016.12.011 28139362

[28] Finkelstein SE Timmerman R McBride WH Schaue D Hoffe SE Mantz CA. The confluence of stereotactic ablative radiotherapy and tumor immunology. Clin Dev Immunol. (2011) 2011:439752. 10.1155/2011/439752 22162711 PMC3227385

[29] Muraro E Furlan C Avanzo M Martorelli D Comaro E Rizzo A. Local high-dose radiotherapy induces systemic immunomodulating effects of potential therapeutic relevance in oligometastatic breast cancer. Front Immunol. (2017) 8:1476. 10.3389/fimmu.2017.01476 29163540 PMC5681493

[30] Taylor CW Kirby AM. Cardiac side-effects from breast cancer radiotherapy. Clin Oncol. (2015) 27:621–9. 10.1016/j.clon.2015.06.007 26133462

[31] Schafer R Strnad V Polgar C Uter W Hildebrandt G Ott OJ Quality-of-life results for accelerated partial breast cancer irradiation with interstitial brachytherapy versus whole-breast irradiation in early breast cancer after breast-conserving surgery (GEC-ESTRO): 5-year results of a randomized, phase 3 trial. Lancet Oncol. (2018) 19:834–44. 10.1016/S1470-2045(18)30195-5 29695348

[32] Thomas JS Hanby AM Russell N van Tienhoven G Riddle K Anderson N. The BIG 2.04 MRC/EORTC SUPREMO Trial: pathology quality assurance of a large phase 3 randomised international clinical trial of postmastectomy radiotherapy in intermediate-risk breast cancer. Breast Cancer Res Treat. (2017) 163:63–9. 10.1007/s10549-017-4145-4 28190252 PMC5387007

