# Supplementary material for: Post-mastectomy Radiotherapy in T1-2 Breast Cancer Patients With One to Three Lymph Node Metastases: A Propensity Score Matching Analysis
Source: Front Oncol. 2020 Feb 14;9:1551. doi: 10.3389/fonc.2019.01551 (PMC7033474; doi:10.3389/fonc.2019.01551)
Supplement: Supplementary file 1 [file Data_Sheet_1.pdf]

Supplementary Material

Supplementary Table 1. Baseline features in patients with one lymph node positive between PMRT group and no-PMRT group

Variable	No. of Patients (%)		*P*
	PMRT group (*N*=5590)	no-PMRT group (*N*=5545)	
Age at diagnosis (years)			0.977
<40	879(15.72)	878(15.83)	
40-70	4076(72.92)	4033(72.73)	
≥70	635(11.36)	634(11.43)	
Race			0.977
White	4303(76.98)	4264(76.90)	
Black	678(12.13)	670(12.08)	
Other*	609(10.89)	611(11.02)	
Marital status			0.982
Married	3616(64.69)	3588(64.71)	
Unmarried#	1974(35.31)	1957(35.29)	
Tumor stage			0.827
T1	1991(35.62)	1986(35.82)	
T2	3599(64.38)	3559(64.18)	
Grade			0.842
1	592(10.59)	594(10.71)	
2	2239(40.05)	2245(40.49)	
3†	2759(49.36)	2706(48.80)	
ER status			0.356
Negative	1322(23.65)	1261(22.74)	
Positive	4268(76.35)	4284(77.26)	
PR status			0.271
Negative	1970(35.24)	1899(34.25)	
Positive	3620(64.76)	3646(65.75)	
Chemotherapy			0.817
No	822(14.70)	824(14.86)	
Yes	4768(85.30)	4721(85.14)	
Specific death	559(10.00)	539(9.72)	0.621

** including American Indian/AK Native and Asian/Pacific Islander; # including single, separated, divorced, widowed and unmarried or domestic partner; † including Poorly differentiated and undifferentiated; PMRT, post-mastectomy radiotherapy; ER, estrogen receptor; PR, progesterone receptor; LN, lymph node.*

Supplementary Table 2. Baseline features in patients with two lymph nodes positive between PMRT group and no-PMRT group

**Variable**	**No. of Patients (%)**		***P***
	**PMRT group (*N*=3923)**	**no-PMRT group (*N*=4276)**	
Age at diagnosis (years)			0.034
<40	570(14.53)	559(13.07)	
40-70	2901(73.95)	3268(76.43)	
≥70	452(11.52)	449(10.50)	
Race			0.183
White	3097(78.94)	3349(78.32)	
Black	441(11.24)	457(10.69)	
Other*	385(9,81)	470(10.99)	
Marital status			0.700
Married	2564(65.36)	2812(65.76)	
Unmarried#	1359(34.64)	1464(32.24)	
Tumor stage			0.001
T1	1277(32.55)	1252(29.28)	
T2	2646(67.45)	3024(70.72)	
Grade			0.053
1	334(17.12)	326(7.62)	
2	1712(43.64)	1797(42.03)	
3†	1877(47.85)	2153(50.35)	
ER status			0.591
Negative	835(21.28)	931(21.77)	
Positive	3088(78.72)	3345(78.23)	
PR status			0.483
Negative	1235(31.48)	1377(32.20)	
Positive	2688(68.52)	2899(67.80)	
Chemotherapy			0.185
No	463(11.80)	465(10.87)	
Yes	3460(88.20)	3811(89.13)	
Specific death	418(10.66)	538(12.58)	0.007

** including American Indian/AK Native and Asian/Pacific Islander; # including single, separated, divorced, widowed and unmarried or domestic partner; † including Poorly differentiated and undifferentiated; PMRT, post-mastectomy radiotherapy; ER, estrogen receptor; PR, progesterone receptor; LN, lymph node.*

Supplementary Table 3. Baseline features in patients with three lymph nodes positive between PMRT group and no-PMRT group

**Variable**	**No. of Patients (%)**		***P***
	**PMRT group (*N*=3072)**	**no-PMRT group (*N*=2764)**	
Age at diagnosis (years)			<0.001
<40	436(14.19)	317(11.47)	
40-70	2284(74.35)	2043(73.91)	
≥70	352(11.46)	404(14.62)	
Race			0.210
White	2382(77.54)	2190(79.23)	
Black	364(11.85)	316(11.43)	
Other*	326(10.61)	258(9.33)	
Marital status			0.711
Married	2007(65.33)	1793(64.87)	
Unmarried#	1065(34.67)	971(35.13)	
Tumor stage			0.001
T1	920(29.95)	938(33.94)	
T2	2152(70.05)	1826(66.06)	
Grade			0.245
1	225(7.78)	229(8.29)	
2	1310(42.64)	1200(43.42)	
3†	1537(50.03)	1335(48.30)	
ER status			0.664
Negative	697(22.69)	614(22.21)	
Positive	2375(77.31)	2150(77.79)	
PR status			0.917
Negative	1013(32.98)	915(33.10)	
Positive	2059(67.02)	1849(66.90)	
Chemotherapy			0.211
No	306(9.96)	303(10.96)	
Yes	2766(90.04)	2461(89.04)	
Specific death	398(12.96)	465(16.82)	<0.001

** including American Indian/AK Native and Asian/Pacific Islander; # including single, separated, divorced, widowed and unmarried or domestic partner; † including Poorly differentiated and undifferentiated; PMRT, post-mastectomy radiotherapy; ER, estrogen receptor; PR, progesterone receptor; LN, lymph node.*
